# Health systems context(s) for integrating mental health into primary health care in six Emerald countries: a situation analysis

**DOI:** 10.1186/s13033-016-0114-2

**Published:** 2017-01-05

**Authors:** James Mugisha, Jibril Abdulmalik, Charlotte Hanlon, Inge Petersen, Crick Lund, Nawaraj Upadhaya, Shalini Ahuja, Rahul Shidhaye, Ntokozo Mntambo, Atalay Alem, Oye Gureje, Fred Kigozi

**Affiliations:** 1 Kyambogo University, Kampala, Uganda; 2 Butabika Hospital Emerald Project, Kampala, Uganda; 3 Stellenbosch University, Stellenbosch, South Africa; 4 Department of Psychiatry, WHO Collaborating Centre for Research and Training in Mental Health and Neuroscience, University of Ibadan, Ibadan, Nigeria; 5 College of Health Sciences, School of Medicine, Department of Psychiatry, Addis Ababa University, Addis Ababa, Ethiopia; 6 Centre for Global Mental Health, Institute of Psychiatry, Psychology and Neuroscience, King’s College London, London, UK; 7 EMERALD Project, School of Applied Human Sciences, University of KwaZulu-Natal, Durban, South Africa; 8 Alan J Flisher Centre for Public Mental Health, Department of Psychiatry and Mental Health, University of Cape Town, 46 Sawkins Road, Rondebosch, Cape Town, 7700 South Africa; 9 Transcultural Psychosocial Organization (TPO) Nepal, Baluwatar, Kathmandu, Nepal; 10 Public Health Foundation of India, Gurgaon, India

## Abstract

**Background:**

Mental, neurological and substance use disorders contribute to a significant proportion of the world’s disease burden, including in low and middle income countries (LMICs). In this study, we focused on the health systems required to support integration of mental health into primary health care (PHC) in Ethiopia, India, Nepal, Nigeria, South Africa and Uganda.

**Methods:**

A checklist guided by the World Health Organization Assessment Instrument for Mental Health Systems (WHO-AIMS) was developed and was used for data collection in each of the six countries participating in the Emerging mental health systems in low and middle-income countries (Emerald) research consortium. The documents reviewed were from the following domains: mental health legislation, health policies/plans and relevant country health programs. Data were analyzed using thematic content analysis.

**Results:**

Three of the study countries (Ethiopia, Nepal, Nigeria, and Uganda) were working towards developing mental health legislation. South Africa and India were ahead of other countries, having enacted recent Mental Health Care Act in 2004 and 2016, respectively. Among all the 6 study countries, only Nepal, Nigeria and South Africa had a standalone mental health policy. However, other countries had related health policies where mental health was mentioned. The lack of fully fledged policies is likely to limit opportunities for resource mobilization for the mental health sector and efforts to integrate mental health into PHC. Most countries were found to be allocating inadequate budgets from the health budget for mental health, with South Africa (5%) and Nepal (0.17%) were the countries with the highest and lowest proportions of health budgets spent on mental health, respectively. Other vital resources that support integration such as human resources and health facilities for mental health services were found to be in adequate in all the study countries. Monitoring and evaluation systems to support the integration of mental health into PHC in all the study countries were also inadequate.

**Conclusion:**

Integration of mental health into PHC will require addressing the resource limitations that have been identified in this study. There is a need for up to date mental health legislation and policies to engender commitment in allocating resources to mental health services.

**Electronic supplementary material:**

The online version of this article (doi:10.1186/s13033-016-0114-2) contains supplementary material, which is available to authorized users.

## Background

Mental disorders constitute a substantial and growing disease burden globally [1, 2]. In 2010, about 10% of the global burden of disease is attributed to neuropsychiatric disorders, mostly due to the high prevalence and chronicity of the more commonly occurring mental disorders [3]. Despite the growing burden of mental illness, mental health services remain a low priority in most low and middle income countries (LMICs), where greater attention is given to the control and eradication of infectious diseases as well as to conditions associated with reproductive, maternal and child health [1, 4]. This may be understandable, due to the high mortalities and morbidities that are directly associated with these priority conditions, [1, 4, 5] but it translates into neglect and a lack of access to care, for the increasing population with mental health conditions in LMICs.

In response to the challenges posed by the large burden attributable to mental disorders, there is now a growing global interest to design and evaluate strategies that can effectively help countries scale up mental health services for their populations [6–8]. In this respect, several authors [6–9] have argued that the integration of mental health into primary health care (PHC) is one of the fundamental strategies necessary to provide the full spectrum of mental health care, consisting of prevention and health promotion, early intervention and rehabilitation. A few studies [6, 10] have assessed factors that may facilitate or hinder the goal of integrating mental health into PHC. However, the data presented in these studies are derived mostly from large-scale global studies and therefore present difficulties in delineating country specific potentialities and constraints relating to integrating mental health into PHC in LMICs.

In this study, we undertook an assessment of the existing system level resources for integrating mental health into PHC in six LMICs participating in the Emerging mental health systems in low and middle-income countries (Emerald) project: Ethiopia, India, Nepal, Nigeria, South Africa and Uganda. The Emerald project aims to identify key health system barriers, and to proffer evidence-based solutions for the scaled-up delivery of mental health services in LMICs, and by doing so, ultimately improve mental health outcomes in a fair and efficient way [11]. Specifically, the project aims to: (a) establish adequate, fair and sustainable resourcing of mental health care, (b) enhance access to integrated community-based mental health care, and (c) improve coverage of care and cost-effective care to reduce disease burden and the economic impact of mental disorders. In each of the Emerald countries there are efforts underway to implement and scale-up integration of mental health into PHC.

## Methods

### Study countries

All study countries reported to be under democratic political systems. Ethiopia, Nepal and Uganda are classified as low income countries with population and gross domestic product (GDP) of just under 100 million people and 61 billion US dollars (Ethiopia); 28.4 million people and 60.4 billion US dollars (Nepal); while Uganda has 39 million people and 23.6 billion US, dollars respectively. The two countries of India and Nigeria are classified as lower middle income countries with respective population and GDP of 1.31 billion and 2.07 Trillion US Dollars (India); and 182 million and 481.07 billion US Dollars (Nigeria). South Africa is classified as an upper middle income country, with a population of 55 million and a GDP of 312.80 billion US dollars. Most of the health systems in the study countries are overstretched by an overall high burden of disease amidst limited resources [11]. Some of the study countries have some pockets of civil conflict (Nigeria) while others are emerging from conflict (Uganda, Nepal). The numerous indicators of development of the study countries are summarized in Table 1.

**Table 1:** Indicators of development of the six Emerald study countries

	Ethiopia	India	Nepal	Nigeria	Uganda	South Africa
Population density	94.1 million	1.3 billion people	28.4 million	180,000,000	34.6 million	Population: 55 million
Government system	*Federal Democratic Republic*	Exists Democratic governance	Multiparty democracy	Democracy, with a Federation system	Democracy with a decentralized system at local government level	
GDP	61.54 billion current usd	$2.25 trillion nominal; 2016	60.4 billion dollars	415.08 billion US dollars	$26.369 billion current usd (http://data.worldbankorg/indicator/NY.GDP.MKTP.CD?locations=UG)	Constitutional democracy with a three-tier system of government and an independent judiciary
HDI	0.442	0.609	0.548	0.504	0.483	312.8 billion USD
MH Law	None	Present, MH Care Bill 2013	Drafted but not yet endorsed	Draft undergoing legislative review	Yes, 1964	Mental Health Care Act (No. 17 of 2002)
MH policy and plan	Integrated into health plan specific National Mental Health Strategy	MH policy 2014, MH plan 365 2014	Mental health policy 1996. There is no mental health plan.	Revised policy 2013; plan under preparation.	No, standalone (2012)	National Mental Health Policy Framework and Strategic Plan 2013–2020
MH Budget (USD)	Not available	Not available	Not available	No explicit MH budget	Not available	There is no specific budget for mental health at national or provincial level. Mental health services are funded out of general health budgets
Percentage of MH budget compared to Health budget	0.9	0.06%	0.17%	3.3%	0.9	Annually, 4% of the health care budget is spent on mental health
Number of psychiatrists (/100,000 population)	0.05	0.07	0.13	0.1	0.09	0.28
Number of psychiatric nurse (/100,000 population)	0.58	0.12	0.27	0.7		10.08
Number of doctors (/100,000 population)	0.022/1000	36/100,000 population (allopathic doctors)	4.9 0.21/1000	0.4/1000	0.117 (per 1000)	0.45
Number of nurses (/100,000 population)	Not available	1010/100,000	Not available	1.6	Not available	807 nurses to 1 patient (data not available per 100,000 population)
Number of mental hospital	1	43	1	8	1	23 public mental hospitals
Number of psychiatric department in General Hospital	11	10,000	17	28	14	41 psychiatric inpatient units
Number of General Hospital	311	–	Not available	825	155	700
Number of psychiatric beds/100,000 population	0.6	1.46	1.	1.3	2.77	18.0

### Data collection

A qualitative document review approach was adopted by this study. The documents reviewed were purposively identified on the basis of providing information on vital building blocks of a health system, as defined in the World Health Organization Assessment Instrument for Mental Health Systems (WHO-AIMS), [12]. These include: mental health legislation, mental health policies and plans, general health policies that included mental health into general health policy, financing, human resources, range and availability of mental health services in the country, integration of mental health into information, education and communication (IEC); synergies among HIV/AIDS and mental health, maternal health care and mental health. In addition, other resources include, integration of mental health into general hospital services, equity in relation to existing policies, monitoring and evaluation, mental health rights and benefits. The tool used to review these resources was a checklist based on the themes identified in the WHO-AIMS [12] and modified to suit the country contexts (see Additional file 1: Appendix). In this study, we depended on purposively selected grey literature because little scientific evidence exists in this field for LMICs in general, and in the study countries in particular. Review of documents was cross-sectional as a way of ascertaining the current status of resources available for integration of mental health into PHC. In essence, different countries were at different stages of developing mental health resources. In some study countries (such as Uganda) some of the vital documents that were included in this review were identified by contacting senior managers at the Ministry of Health. Each country’s research team conducted a review of policy documents, plans, legislative frameworks and other relevant program documents that were available at the Ministry/Department of Health. Some of the vital documents reviewed in the study countries included: mental health bills/acts, health policies and strategic plans, mental health policies and plans (where available), Ministry of health budgets, human resources plan and staff deployments, ethical guidelines (e.g. for research), monitoring and evaluation plans, program/sector performance reports, among others. The specific documents that were reviewed in each study country are summarized in Table 2.

**Table 2:** Documents reviewed by each study country

Domain	Name/type of document	Author and year
Uganda		
*Domain one* Mental health legislation	Uganda Mental Health Treatment Act 1964 Uganda Draft Mental Health Bill (2012)	Uganda Ministry of Justice, Kampala, Uganda Uganda Ministry of Justice and Constitutional Affairs Kampala, Uganda
*Domain two* Mental health policy	Uganda Mental Health Neurological and Substance Use Policy (2011) Uganda National Health Policy (2010)	Uganda Ministry of Health Kampala, Uganda Uganda Ministry of Health Kampala, Uganda
*Domain three* Financing	Uganda Health Sector Strategic Plan III (2010/11–2014/15) Uganda Second National Development Plan 201011–201415/ http://hdr.undp.org/en/countries/profiles	Uganda Ministry of Health Kampala, Uganda Uganda National Planning Authority, Kampala, Uganda
*Domain four* Human resources	Uganda Health Sector Strategic Plan III (2010/11–2014/15) http://www.aho.afro.who.int/profiles_information http://data.worldbank.org/indicator	Uganda Ministry of Health Kampala, Uganda
*Domain five* Mental health services	Uganda Health Sector Strategic Plan III (2010/11–2014/15) Uganda Mental Health Neurological and Substance Use Policy (2011)	Uganda Ministry of Health Kampala, Uganda Uganda Ministry of Health Kampala, Uganda
*Domain six* Integration of mental health into Information, education and communication systems (IEC)	Uganda Mental Health Neurological and Substance Use Policy (2011)	Uganda Ministry of Health Kampala, Uganda
*Domain seven* HIV/AIDS mental health	Uganda Health Sector Strategic Plan III (2010/11–2014/15) Uganda Mental Health Neurological and Substance Use Policy (2011)	Uganda Ministry of Health Kampala, Uganda Uganda Ministry of Health Kampala, Uganda
*Domain eight* Integration of mental health into general health services	Uganda Health Sector Strategic Plan III (2010/11–2014/15)	Uganda Ministry of Health Kampala, Uganda
*Domain nine* Issues of equity in relation to existing policies	Uganda Ministry of Health Kampala, Uganda	Uganda Ministry of Health Kampala, Uganda
*Monitoring and evaluation ten* any other documents	Uganda Ministry of Health Kampala, Uganda	Uganda Ministry of Health Kampala, Uganda
Ethiopia		
*Domain one* Mental health legislation	National Mental Health Strategy (2011/2012–2015/2016)	Federal Ministry of Health of Ethiopia
*Domain two* Mental health policy	National Mental Health Strategy (2011/2012–2015/2016)	Federal Ministry of Health of Ethiopia
*Domain three* Financing	Health Sector Transformation Plan (2015/2016–2019/2020) http://hdr.undp.org/en/countries/profiles	Federal Ministry of Health of Ethiopia
*Domain four* Human resources	Health Sector Transformation Plan (2015/2016–2019/2020) National Mental Health Strategy (2011/2012–2015/2016) http://www.aho.afro.who.int/profiles_information http://data.worldbank.org/indicator	Federal Ministry of Health of Ethiopia
*Domain five* Mental health services		
*Domain six* Integration of mental health into Information, education and communication systems (IEC)	National Mental Health Strategy (2011/2012–2015/2016)	Federal Ministry of Health of Ethiopia
*Domain seven* HIV/AIDS mental health	National Mental Health Strategy (2011/2012–2015/2016)	Federal Democratic Republic of Ethiopia Ministry of Health. Ministry of Health, Ethiopia
*Domain eight* Integration of mental health into general health services	Health Sector Transformation Plan (2015/2016–2019/2020)	Federal Democratic Republic of Ethiopia Ministry of Health. Ministry of Health, Ethiopia
*Domain nine* Issues of equity in relation to existing policies	National Mental Health Strategy 2012/13–2015/16	Federal Democratic Republic of Ethiopia Ministry of Health. Ministry of Health, Ethiopia
*Monitoring and evaluation ten* any other documents	National Mental Health Strategy (2011/2012–2015/2016)	Federal Democratic Republic of Ethiopia Ministry of Health. Ministry of Health, Ethiopia
Nigeria		
*Domain one* Mental health legislation	Expert Opinions	2015
*Domain two* Mental health policy	National Policy for Mental Service Delivery. Fedaral Ministry of Health (FMoH), Abuja, Nigeria	FMoH (2013)
*Domain three* Financing	WHO-AIMS Country Report	World Health Organisation (2003), Nigeria
*Domain four* Human resources	World Bank report http://data.worldbank.org/indicator	http://data.worldbank.org/indicator
*Domain five* Mental health services	FMoH, Hospital Services Unit and Expert Reviews	FMoH (2015)
*Domain six* Integration of mental health into Information, education and communication systems (IEC)	Revised National Mental Health Policy of 2013	FMoH (2013), Abuja Nigeria
*Domain seven* HIV/AIDS mental health	Revised National Mental Health Policy of 2013	FMoH (2013)
*Domain eight* Integration of mental health into general health services	Revised National Mental Health Policy of 2013	Revised National Mental Health Policy
*Domain nine* Issues of equity in relation to existing policies	Revised National Mental Health Policy	Revised National Mental Health Policy
*Monitoring and evaluation ten* any other documents	Revised National Mental Health Policy	Revised National Mental Health Policy
South Africa		
*Domain one* Mental health legislation	National Mental Health Policy Framework and Strategic Plan	National Department of Health, South Africa (2013–2020)
	Mental Health Care Act (17 of 2002)	National Department of Health, South Africa
*Domain two* Mental health policy	National Mental Health Policy Framework and Strategic Plan	National Department of Health, South Africa (2013–2020)
*Domain three* Financing		
*Domain four* Human resources		
	WHO interactive charts: Psychiatrists and nurses working in mental health sector	2014
	WHO-AIMS report on mental health system in South Africa	2007
	Article-*Bed/population ratios in South African public sector mental health services.*	Lund, C., Flisher A, J., Porteus K., Lee T. Bed/population ratio in South Africa Public Sector Mental Health Services. Soc Psychiatry Psychiatry Epidemiol. 2002; 37(7), 346–9
	Human resources for health, South Africa: HRH strategy for the health sector (2012/13–2016/17)	National Department of Health, South Africa (2011)
*Domain five* Mental health services		
*Domain six* Integration of mental health into Information, education and communication systems (IEC)	WHO-AIMS report on mental health system in South Africa	2007
	Article-*Bed/population ratios in South African public sector mental health services.*	Lund, C., Flisher A, J., Porteus K., Lee T. Bed/population ratio in South Africa Public Sector Mental Health Services. Soc Psychiatry Psychiatry Epidemiol. 2002; 37(7), 346–9
*Domain seven* HIV/AIDS mental health	National Mental Health Policy Framework and Strategic Plan	National Department of Health, South Africa (2013–2020)
*Domain eight* Integration of mental health into general health services	National Mental Health Policy Framework and Strategic Plan	National Department of Health, South Africa (2013–2020)
*Domain nine* Issues of equity in relation to existing policies	National Mental Health Policy Framework and Strategic Plan	National Department of Health, South Africa (2013–2020)
*Monitoring and evaluation ten* any other documents	National Mental Health Policy Framework and Strategic Plan	National Department of Health, South Africa (2013–2020)
India		
*Domain one* Mental health legislation	Mental Health Bill 2013 Mental Health Bill 1987	Government of India
*Domain two* Mental health policy	Mental Health Policy 2014	Mental Health Policy Group, MOHFW
*Domain three* Financing	WHO mental health financing, 2003	Funk, M., Saraceno, B., Drew, N., Faydi., E. Integrating mental health into primary healthcare. Ment Health Fam Med. 2008 Mar; 5(1): 5–8.
*Domain four* Human resources	International Minstry of Health reports	Ministry of Health
*Domain five* Mental health services		
*Domain six* Integration of mental health into Information, education and communication systems (IEC)	Mental Health Plan 365	Ministry of Health and Family Welfare (2014)
*Domain seven* HIV/AIDS mental health	Mental Health Policy 2014	Ministry of Health and Family Welfare (2014)
*Domain eight* Integration of mental health into general health services	Mental Health Policy 2014	Ministry of Health and Family Welfare (2014)
*Domain nine* Issues of equity in relation to existing policies	Mental Health Policy 2014	Ministry of Health and Family Welfare (2014)
*Monitoring and evaluation ten* any other documents	Mental Health Plan 365	Ministry of Health and Family Welfare (2014)
Nepal		
*Domain one* Mental health legislation	Draft mental health treatment and protection act 2006.	Ministry of Health (2006)
*Domain two* Mental health policy	Mental Health Policy 1996	Ministry of Health (1996)
*Domain three* Financing	Jha et al. (2009) Mental Health Service in Nepal. Journal of Nepal Medical Association: Vol. 48 No.2 Issue 174 WHO and Ministry of Health, Nepal, 2006. WHO AIMS Report on Mental Health Systems in Nepal. World Health Organization and Ministry of Health, Kathmandu, Nepal	Jha et. Al (2009) Mental Health Service in Nepal. Journal of Nepal Medical Association: Vol. 48 No.2 Issue 174
*Domain four* Human resources	WHO and Ministry of Health, Nepal, 2006. WHO AIMS Report on Mental Health Systems in Nepal. World Health Organization and Ministry of Health, Kathmandu, Nepal http://www.aho.afro.who.int/profiles_information http://data.worldbank.org/indicator	WHO and Ministry of Health Nepal 2006
*Domain five* Mental health services	National Health Sector Support Program (NHSSP) II: 2010–2015.Ministry of Health and Population, Kathmandu, Nepal. WHO and Ministry of Health, Nepal, 2006. WHO AIMS Report on Mental Health Systems in Nepal. World Health Organization and Ministry of Health, Kathmandu, Nepal	Ministry of Health [20]
*Domain six* Integration of mental health into Information, education and communication systems (IEC)	Not available	–
*Domain seven* HIV/AIDS mental health	Not available	–
*Domain eight* Integration of mental health into general health services	Second long term health plan (1997–2017 and National Health Sector Support Program (NHSSP) II: 2010–2015 The National Health Policy of 1991	Ministry of Health (1997)
*Domain nine* Issues of equity in relation to existing policies	Not available	
*Monitoring and evaluation ten* any other documents	Department of Health Services (DoHS) annual reports, 2008/09, 2009/10, 2010/11	Department of Health Services (DoHS) annual reports, 2008/09, 2009/10, 2010/11
	NGO reports and informal discussion with relevant officials at the ministry of health and department of health services	

### Data analysis

All the data collected from the study sites were summarized in a matrix. Content thematic analysis was used to analyze the data. A priori themes comprised the pre-conceived categories from the WHO-AIMS (12), with subcategories and emerging themes were developed under each category.

### Ethics statement

All study sites had secured ethical approval from their respective ethical boards and this research project was one of the ongoing Emerald project activities. Ethics approval was also obtained from King’s College London and the WHO.

## Results

Under this section, we present our results based on some of the overarching WHO-AIMS categories of the health system that the study investigated.

### Mental health legislation and human rights

In terms of legislation, South Africa has the Mental Health Care Act No. 17 of 2002 [13]. India has a Mental Health Care Act, 2016. Nigeria has the Nigerian Mental Health Bill (2013) which is undergoing consideration by the country’s National Assembly. Currently however, it is the old Lunacy Act of 1958 that still exists in the country. In Uganda, a Mental Health Bill was produced in 2009. A revised version was produced in 2011 and it is still before the cabinet.

Nepal has a draft Mental Health Bill (Treatment and Protection) Act 2006 [14] which was revised in 2012 but has still not been passed by the parliament. There is also the Disabled Welfare and Protection Act, 1982 and The Protection and Welfare of the Disabled Persons Rules, 1994. In Ethiopia, dedicated mental health legislation does not exist but is currently under development. This movement towards development of new legislations in some of the study countries is inconformity with the provisions of WHO which endorsed mental health as a universal human right and a fundamental goal for health care systems of all countries [15], as evident from the content of the newer legislations.

In terms of human rights, the draft mental health bill of Nepal, has provisions for managing patients who require treatment against their will. In South Africa, the Mental Health Care Act of 2000 [13] provides that designated general hospitals are required to admit and assess people who are admitted involuntarily with psychiatric emergencies for a minimum of 72-h before they may be referred to psychiatric hospitals. If after a 72-h observation, a patient requires in-patient treatment they must be admitted to a specialist psychiatric ward or hospital. Review Boards in each province oversee involuntary admission and related appeals. In Uganda, the old law and the current draft mental treatment Act (2011), have protocols for managing patients who require treatment against their will; replacing the old “Mental Treatment Act of 1938 (Ch 279)”, amended 1964 (section 10). As Uganda awaits the passage of the new law by cabinet and parliament, the old protocol is still being utilized to administer treatments to patients against their will. While Nigeria similarly awaits the passage of its new mental health bill, sections 10–13 of Nigeria’s old Lunacy Act permits involuntary hospitalization for less than 7 days and requires a Magistrate’s order if it is longer than 7 days. The mental health bills of South Africa and India and the draft mental health Bills of Uganda, Nepal, Nigeria are aligned to the United Nations Convention on the Rights of People with Disability (CRPD), and most of the issues on human rights are also inherent in the national Constitutions of the study countries.

#### Mental health policy

Mental health policies provide a framework for mental health system development [3]. The policy largely conveys government’s commitments organized in a set of values, principles, objectives and areas for action to improve the mental health of a population [4]. Based on the findings of the documents review, South Africa has a new Mental Health Policy and Strategic Plan (2013–2020) [16]. The South African policy aims at transforming mental health services and ensuring that quality mental health services are accessible, equitable, and comprehensive, and are integrated at all levels of the health system. This policy is aligned to the WHO Mental Health Action Plan that provides for task shifting and the integration of mental health into primary health care services [16]. The policy also integrates scientific evidence and best practices with an emphasis on human rights and vulnerable populations [16]. Nepal has a Mental Health Policy (1996) [17] which has not been implemented for over 15 years. There is also no mental health desk in the Ministry of Health and Population in Nepal. At the time of this review, Uganda had a draft mental health policy [18] which was before the Ministry of Health’s top management for approval. India’s first mental health policy was finally released in October 2014. It was spearheaded by the Ministry of Health and Family Welfare which had constituted a policy group consisting of academics, psychiatrists, psychologists, service user representatives and representatives from the ministry. In Ethiopia, the policy context is such that there are no disease specific policies. Instead, the country has an overarching health policy and each condition is targeted through a policy strategy. Ethiopia has a National Mental Health Strategy (2012–2016) which provides policy direction, in the absence of a formal policy. In Nigeria, a National Mental Health Policy was first developed in 1991, and has recently been revised in 2013. The South African and Nigerian mental health policies, and the draft mental health policy for Uganda are aligned to the WHO Mental Health Action Plan because of their focus on promotion of human rights, provisions for participation of people/stakeholders in policy development, and a focus on advocacy, promotion, prevention and rehabilitation of those with mental disorders (among others) as key elements of a functional mental health policy.

### Mental health plans

Mental health plans are essential for guiding the activities that have to be implemented to meet policy objectives and typically include vital elements such as budgets and time frames [4]. South Africa has a Mental Health Action Plan [16] which provides a roadmap for the implementation of the mental policy. Nigeria’s mental health action plan is being developed. In the rest of the study countries, mental health is directly mentioned in some of the strategic plans in the general health sector. For example, in Ethiopia, it is mentioned in the Health Sector Transformation Plan (2015/2016–2019/2020), within the domain of prevention and control of Non-communicable diseases (NCDs). The Ethiopia health plan includes a target to make mental health services available in every district in the country by 2020. In India, the National Mental Health Program is a comprehensive program which includes plans to deliver community-based mental health care in 100 districts all over the country [19]. There were also efforts towards modernization of state-run mental hospitals; upgrading of psychiatry wings in the government medical colleges and general hospitals; Information, Education and Communication (IEC) activities; as well as research and training in mental health for improving service delivery. In Nepal, mental health is part of the essential health care services in the government’s second long term Health Plan (1997–2017) and the National Health Sector Support Program (NHSSP II 2010–2015). In Uganda, mental health is under the section on “prevention and control of non-communicable diseases (NCDs), disabilities and injuries” in the general health policy as well as the National Health Sector Investment Plan (1999/2000–2009/10) [20]. Uganda, Ethiopia and Nepal do not comprehensively define the objectives, activities and indicators of success relating to mental health in national plans where mental health is placed. Also, key elements such as community involvement, advocacy, user involvement in mental health service delivery among others; are missing.

At district/regional level, the health plans of Uganda and Nigeria do not specifically mention mental health. However, in the two study countries mentioned above, integrated mental health packages are delivered through the pilot implementation of the WHO’s Mental health gap action programme (mhGAP) in selected districts [21]. The mhGAP project is meant to provide lessons for further integration of mental health in other districts. In Ethiopia, the Federal Ministry of Health is scaling up mental health care integrated into primary care. Memoranda of Understanding have been signed with the Regional Health Bureaus and a dedicated budget availed to support the scale-up plan.

In South Africa, at the district level, mental health is specifically mentioned in the program for “Integration of mental health into PHC” and district guidelines have been developed [22]. In Nepal, the National Health Policy (1991) devolves mental health delivery to regional hospitals where specialized mental health services are provided. In Ethiopia, there is the district-based planning which takes the Ministry of Health plan as the starting point but may adapt to local conditions. Mechanisms for coordination at the district level exist within the national strategic plans in Ethiopia, Uganda and South Africa and this creates opportunity for integration of mental health into PHC. In Nigeria, there is no systematic mental health activities going on at the district and primary health care level, except where this is occurring in the context of a research project.

### Financing

The volume of funds allocated for mental health service delivery can facilitate or hinder integration of these services into PHC. The volume of financial resources available in the different countries for mental health is summarized in Table 1. Among all the study countries, Nepal spends the lowest percentage (0.06%) of the health budget on mental health while South Africa spends the highest percentage (5%) of its health budget on mental health. Given the size of the budgets allocated to the mental health sector in all the study countries, it is unlikely that adequate and quality health services can be provided. Private sector contributions are not reflected in the existing plans reviewed and are difficult to assess. However, it is unlikely that the high poverty levels in most of the study countries can allow adequate private contributions to bridge the gap in financing the mental health sector.

### Human resources

The human resources available in the health system to support integration of mental health into PHC are summarized in Table 1. Documents reviewed during this study indicated that South Africa has 0.28 per 100,000 populations Uganda has 0.09 psychiatrists per 100,000 populations. Nigeria has 0.1 per 100,000 populations; India and Ethiopia have 0.07 psychiatrists per 100,000 populations while Nepal has 0.13 per 100,000 populations. Our findings above indicate that the number of psychiatrists in relation to the population of the study countries is still unacceptably low. The number of other critical cadres such as psychiatric nurses were also insufficient (Table 1).

### Mental health services

As indicated in Table 1, in terms of mental health services, South Africa has 23 mental hospitals and 41 psychiatric units in the general hospitals. Uganda has 1 mental hospital, and 16 units in general hospitals; while Nigeria has 8 mental hospitals, and 28 units in general hospitals. India has 43 mental hospitals and 10,000 units in general hospitals; while Nepal has 1 mental hospital and 17 units in general hospitals. The number of psychiatric beds per 100,000 population was also insufficient in all countries (Table 1).

### Integration of mental health into information, education and communication (IEC) programs

In Ethiopia, the national mental health strategy has programs specified and some are related to IEC. In Nepal and Uganda, integration of mental health into IEC is not explicitly stated. For Nigeria and South Africa, National Mental health policies include integration of mental health into information, education and communication and set the specific indicators. No integration of mental health into information, education and communication programs was reported at district/regional level in all the study countries. It is however, important to note that even when integration of IEC is stated in the policy framework(s), IEC programs might not exist in the study countries.

### HIV/AIDS mental health

In Nepal and Nigeria, the general mental health policy does not directly focus on HIV and mental health. In Ethiopia, Uganda and South Africa, people living with HIV and AIDS are identified as a vulnerable group needing targeted mental health interventions.

### Maternal mental health

It is only South Africa where treatment programs for maternal mental health are specifically mentioned in the mental health policy. The new policy of India also focuses on maternal mental health as a sector. It emphasizes the need to increase access to mental health services along with child and reproductive health services. There is no specific mention of maternal mental health in the National Mental Health Policy of Nepal and Nigeria. In Ethiopia, maternal mental health is mentioned under vulnerable groups in the National Mental Health Strategy.

### Integration of mental health into general health services

Review of national health plans and program reports indicated that there is limited provision for integration of mental health in general health services in all study countries. Though mental health care is part of the pre-service training for most health workers in the study countries, there is no uniform in-service training in any of the countries. Levels of skill to manage mental health issues were reported to be low at district levels in all the study districts. Most study districts had low level mental health cadres (for example nurses). There are in-service training opportunities in selected districts (sites) to facilitate the integration of mental health into primary health care (public health centers) using the mhGAP Intervention Guide training module (http://www.who.int/mental_health/mhgap/en/). However, no comprehensive evaluation reports are available so far on the impact of these trainings in the study countries with the exception of Nigeria [22].

### Issues of equity in relation to existing policies

In terms of equity, the South Africa mental health policy and the draft Uganda mental health policy recognize gender issues in mental health service provision. Nigeria’s mental health policy recognizes women under the category of the disadvantaged, requiring special care. In Nepal and India, no gender related issues are directly addressed in the existing general health policy. In Ethiopia, perinatal mothers with mental health problems are recognized as a special group, and for Uganda, all women (perinatal mothers inclusive) are mentioned among the vulnerable groups in the draft mental health policy.

In Uganda and South Africa, the existing mental health policies are linked to poverty reduction and the poor are a special category to be targeted. Similarly, in the rest of the study sites, the poor are targeted under the general health policies. No policy or strategy explicitly addresses issues of equity in relation to rural/urban residence in any EMERALD country.

In South Africa, disability issues are addressed in the mental health policy. In Uganda, Nigeria, India, and Nepal, disability is classified under disadvantaged groups or groups with special needs in the general mental health policy. In Ethiopia, it is not specified in the disadvantaged groups or those identified with special needs. For South Africa, a disability grant is available nationally for persons with physical or mental disability that renders person unfit for work for a period longer than 6 months. Furthermore, there is a strategy to address vulnerable members of society including children and the disabled to promote integration into workplace and communities and enhance skills development to promote self-worth and enhance quality of life. However, there is no evaluation as to whether these services are equitable in the study countries, where they are available.

### Monitoring and evaluation

The National Health Management Information System (HMIS) system of Nepal, South Africa, Ethiopia and Uganda capture mental health indicators; but the HMIS of India and Nigeria do not have mental health indicators. The content of each HMIS for mental health is detailed in our paper on indicators for routine monitoring of effective mental healthcare coverage in low- and middle-income settings: a Delphi study (Mark Jordan).

There are however challenges in study countries about the quality of indicators used to capture mental health issues. For example, in South Africa the mental health indicators at PHC level are only two: numbers screened and numbers treated. These do not help with tracking identification and management of specific disorders where diagnosis and severity would be helpful.

## Discussion

This study contributes to the understanding of resources for integrating mental health into health systems in Emerald countries. It provides important data to inform current and future strategies to respond to the high burden of mental, neurological and substance use disorders (MNS) and planning for the integration of mental health into PHC in the study countries. The study provides a detailed overview of some of the resources available within the essential building blocks of the health system in the study countries.

It has been noted that around 25% of the people who attend a primary health care clinic have a diagnosable mental disorder [9]. Many MNS disorders still go untreated in LMICs and a treatment gap of more than 90% has been reported [3]. Health systems can however respond to the high burden of MNS if strengthened [6, 7]. Through integration, there is an opportunity to provide holistic care, patient centered interventions and ensure cost effectiveness in service delivery at Primary Health care level [8]. People can also seek the services near their home (PHC settings) thus keeping their families together and remain productive [10]. Mental health services also delivered within the primary health care setting can minimize stigma [10].

Legislations to some extent indicate the level of commitment to mental health as a human right [22]. Our findings indicate that apart from South Africa; other study countries were largely in the process of enacting mental health legislations that protect the human rights of people with mental disabilities. Mental health legislation provides a legal framework for enforcing policy objectives, and can reinforce integration by legislating for parity between physical and mental health care; by introducing specific provisions promoting de-institutionalisation and the provision of care in primary healthcare settings [22]. For example, South Africa has a 72-h observation period at designated district and regional hospitals. It is through these concrete formal commitments that integration can take place. Other countries were also making positive strides towards enacting the necessary laws on mental health. The major challenge however, is that this process is normally slow. And, in the absence of updated legislations, the study countries rely on obsolete laws. For example, Uganda and Nigeria currently draw on legislations that are decades old. These do not adequately protect the rights of people with mental disabilities and might not be relevant to the rapidly changing contextual challenges faced by these countries today. It would be important that in study countries where the legislations are out of date, the process of their review is expedited in order to protect the rights of people with disabilities and to support the integration of mental health into primary health care. Furthermore, adequate resources should be put in place to implement the legislations on mental health within the context of primary health care. In South Africa, Marais et al. [22] report that there is insufficient training on the Act (Mental Health Care Act of 2002), as well as a lack of clarity on the responsibilities of the different sectors in its operationalization [22]. Also, as seen from Nepal, the legislation has not been endorsed and implemented for several years even after its drafting in 2006. More advocacy may be needed in this field as these countries continue to make efforts towards integrating mental health into primary health care.

In terms of policy, mental health policies could facilitate strong primary health care delivery as well as integration of mental health into primary health care system [8]. It has been noted that mental health policies in particular can define the specific objectives to be strived for in integrating mental health, while plans can outline in detail the specific strategies and activities required for doing so [8]. South Africa is also more developed than other study countries in terms of having a specific mental health policy framework [16] which operationalizes the aspirations expressed in the legislations. Other study countries seem to be relying on the general health policy and strategic plans. The lack of a fully-fledged mental health policy in some study countries creates some dilemmas: (a) it is difficult to define and attract resources for a sector without a fully-fledged policy, (b) it is difficult to define the required structure and the required resources to deliver the services expected. However, even in South Africa where the specific policy framework exists, there are concerns relating to the slow pace at which the mental health policy is being implemented [22]. It would be important that countries develop and plan for adequate resources to facilitate the development of mental health policies/plans. Wider consultations with key stakeholders will be vital in these processes to foster participatory development of management principles and goals.

It has also been noted that although integrating mental health into PHC is a cost-effective strategy, financial resources are still required to establish and maintain a service at any unit of service delivery [9]. Across countries, health care systems vary in their ability to respond to national health care needs [6]. Many healthcare systems lack the core health system elements needed to provide the most basic set of services. In South Africa, the mental health budget is integrated into PHC but mental health is still sidelined in favor of other priorities and this is likely to stifle or weaken efforts to integrate mental health into primary health care. In other study countries, our findings indicate limited finances for integration of mental health into PHC. The budget allocation cannot match with the increasing number of people with mental illness in the study countries. In order to meet policy imperatives for deinstitutionalization, the scarce resources which historically have been allocated mostly to tertiary institutions are under threat of being re-allocated to PHC centers [9] thus, undermining the continued essential services that tertiary institutions currently provide. Thus, insufficient funding could result in the re-allocation of some of the resources at the national psychiatric hospitals to PHC centers [9] in addition to identifying other resources. There are quite a number of vital activities such as support and supervision, training and incentives for health workers, which carry a minimal budget but are not currently allocated sufficient funds and are therefore out of reach to the communities in the study countries. It is also quite clear that most of the study countries do not have universal health insurance facilities to ensure the affordability of services and full utilization. It is important to note that budget allocations should take care of the disparities in the burden of mental illness in the study countries. Some regions might have unique mental care needs (for example those emerging from civil conflict such as northern Uganda and those in active conflict such as some parts of Nigeria) and integration of mental health into primary health should be sensitive to such contexts.

National governments need to make a more efficient use of the available resources and one of the possible strategies of doing this is to take a phased approach to the integration process [22]. The lessons learned along the way should guide the scale up process in the study countries.

Our findings also indicate that all study countries had insufficient numbers of mental health workers to meet the need for mental health service. This calls for the scaling up of mental health services through its integration into primary health care [23, 24]. Related to this challenge is the ambiguity about the skills set that are required to deliver the integrated packages, though the mhGAPhas apparently tried to solve this dilemma [23]. It has been observed that for any health workforce to be effective, and for care packages to be delivered as intended, treatment guidelines need to be operationalized into coordinated roles and tasks [23]. It is further observed that the starting point for effective integrated care pathways is to specify the necessary skill sets to effectively deliver integrated care and plan [5]. Human resources need to be available to support the delivery of mental health services within the PHC context and if not available they need to be re-directed from tertiary institutions to PHC centers [8]. Another way to handle the human resource gap is to include mental health in the training of undergraduate medical and paramedical students and re-enforce skill development through on the job training as well as through continued support and supervision [8]. Training of primary health workers should also include a component of mental health [8].

Adequate numbers of specialized health care workers are needed for support supervision [8]. These activities should be integrated into other primary health services for sustainability reasons.

### Study limitations

This situation analysis is based purely on documents review. Since mental health is largely given low priority, the level of resources and documentation of mental health resources is low. However, with the support from the senior mental health workers in the study countries, vital documents were secured that have supported this review process. While some countries had new documents in terms of laws and policies, others had quite old legislation and polices. It was therefore difficult to set a uniform time frame for the review due to the above reason. Despite this limitation, study countries were using the old laws and policies to support integration of mental health into primary health care and this forms the major reason for their inclusion into this review. It would have been enriched further by interview data (such as key informant interviews; which were not conducted at that time due to resource limitations). Despite this limitation, this review provides useful information to deepen the understanding of the health systems resources available for integrating of mental health into PHC.

## Conclusion

There is some progress in the study countries on some of the building blocks of the health system that may support the integration of mental health into primary health care. This progress seems to be more visible in the legislations on mental health and to some extent in the policy arena. However, in all the study countries, there are still glaring gaps in the basic building blocks needed to implement the policy and legislative frameworks. Overall, there is a need to critically address the gaps in the resources that could support integration of mental health into PHC in the study countries in order to successfully scale up mental health care services in an accessible and cost-effective manner.

## Additional file

**Additional file 1: Appendix.** Checklist for the overarching themes in the WHO-AIMS study investigated by the study.

## Abbreviations

HIV/AIDS: acquired immune deficiency syndrome
PHC: primary health care
WHO: World Health Organization
mhGAP: mental health gap action programme
mhGAP-IG: mental health gap action programme-intervention guide
