# Supplementary material for: Health systems context(s) for integrating mental health into primary health care in six Emerald countries: a situation analysis
Source: Int J Ment Health Syst. 2017 Jan 5;11:7. doi: 10.1186/s13033-016-0114-2 (PMC5217325; doi:10.1186/s13033-016-0114-2)
Supplement: Supplementary file 1 — Additional file 1: Appendix. Checklist for the overarching themes in the WHO-AIMS study investigated by the study. [file 13033_2016_114_MOESM1_ESM.docx]

**Checklist for the overarching themes in the WHO-AIMS study investigated by the study**

**TABLE I: MENTAL HEALTH POLITICS, POLICIES AND PLANS**

**Political Support**

**National/State Level**

| Questions/themes | **1.1 Political commitment for mental health services (e.g. existence of steering committee/task force at national level, existence of clear guidelines for the task force)** | | | | | |
| --- | --- | --- | --- | --- | --- | --- |
|  | **Countries** | | | | | |
| **National/state Level** | Ethiopia | India | Nepal | Nigeria | South Africa | Uganda |
|  |  |  |  |  |  |  |
| **District level** |  |  |  |  |  |  |
| **Source of Evidence/**  **Date for Data** |  |  |  |  |  |  |
| **Source of Q** |  |  |  |  |  |  |

**TABLE 2:**

**Political Support: National/State Level**

| **Questions/**  **themes** | **1.2. Is mental health specifically mentioned in general health policy / strategic national health plans?** | | | | | |
| --- | --- | --- | --- | --- | --- | --- |
|  | **Countries** | | | | | |
| **National/state Level** | Ethiopia | India | Nepal | Nigeria | South Africa | Uganda |
|  |  |  |  |  |  |  |
| **District level** |  |  |  |  |  |  |
| **Source of Evidence/**  **Date for Data** |  |  |  |  |  |  |
| **Source of Q** |  |  |  |  |  |  |

**Table 3: 2.0 MENTAL HEALTH BUDGET**

| **Questions/**  **themes** | **2.1 Mental health budget as % of total health budget Mental health expenditure as a percentage (%) of overall health expenditure (budgeted Versus actual)** | | | | | |
| --- | --- | --- | --- | --- | --- | --- |
|  | **Countries** | | | | | |
| **National/state Level** | Ethiopia | India | Nepal | Nigeria | South Africa | Uganda |
|  |  |  |  |  |  |  |
| **District level** |  |  |  |  |  |  |
| **Source of Evidence/**  **Date for Data** |  |  |  |  |  |  |
| **Source of Q** |  |  |  |  |  | . |

**Table 4: 3.0.** **MENTAL HEALTH POLICY**

| **Questions/**  **themes** | - 1. **Existence of an officially approved mental health policy / strategy?** | | | | | |
| --- | --- | --- | --- | --- | --- | --- |
|  | **Countries** | | | | | |
| **National/state Level** | Ethiopia | India | Nepal | Nigeria | South Africa | Uganda |
|  |  |  |  |  |  |  |
| **District level** |  |  |  |  |  |  |
| **Source of Evidence/**  **Date for Data** |  |  |  |  |  |  |
| **Source of Q** |  |  |  |  |  |  |

**Table 5: 3.0 MENTAL HEALTH POLICY cont…**

| **Questions/**  **themes** | **3.1.1 If present, what year was mental health policy / strategy last revised?** | | | | | |
| --- | --- | --- | --- | --- | --- | --- |
|  | **Countries** | | | | | |
| **National/**  **state Level** | Ethiopia | India | Nepal | Nigeria | South Africa | Uganda |
|  |  |  |  |  |  |  |
| **District level** |  |  |  |  |  |  |
| **Source of Evidence/**  **Date for Data** |  |  |  |  |  |  |
| **Source of Q** |  |  |  |  |  |  |

**Table 6: 3.0 MENTAL HEALTH POLICY cont…**

| **Questions/**  **themes** | - - 1. **If present, describe extent of the policy / strategy has been implemented? And across how much of the country / state / district?** | | | | | |
| --- | --- | --- | --- | --- | --- | --- |
|  | **Countries** | | | | | |
| **National/**  **state Level** | Ethiopia | India | Nepal | Nigeria | South Africa | Uganda |
|  |  |  |  |  |  |  |
| **District level** |  |  |  |  |  |  |
| **Source of Evidence/**  **Date for Data** |  |  |  |  |  |  |
| **Source of Q** |  |  |  |  |  |  |

**Table 7: 3.0 MENTAL HEALTH POLICY cont…**

| **Questions/**  **themes** | **3.2.0 Does the policy / strategy include:** | | | | | |
| --- | --- | --- | --- | --- | --- | --- |
| **3.2.1** | **Integration of mental health into PHC?** | | | | | |
|  | **Countries** | | | | | |
| **National/state Level** | Ethiopia | India | Nepal | Nigeria | South Africa | Uganda |
|  |  |  |  |  |  |  |
| **District level** |  |  |  |  |  |  |
| **Source of Evidence/**  **Date for Data** |  |  |  |  |  |  |
| **Source of Q** |  |  |  |  |  |  |

**Table 8: 3.0 MENTAL HEALTH POLICY cont…**

| Questions/  themes | **3.2.0 Does the policy / strategy include:** | | | | | |
| --- | --- | --- | --- | --- | --- | --- |
| 3.2.2 | **Integration of mental health into Information, Education and Communication programs?** | | | | | |
|  | **Countries** | | | | | |
| **National/**  **state Level** | Ethiopia | India | Nepal | Nigeria | South Africa | Uganda |
|  |  |  |  |  |  |  |
| **District level** |  |  |  |  |  |  |
| **Source of Evidence/**  **Date for Data** |  |  |  |  |  |  |
| **Source of Q** |  |  |  |  |  |  |

**Table 9: 3.0 MENTAL HEALTH POLICY cont…**

| **Questions/**  **themes** | **3.2.0 Does the policy / strategy include:** | | | | | |
| --- | --- | --- | --- | --- | --- | --- |
| **3.2.3** | **Decentralization to districts?** | | | | | |
|  | **Countries** | | | | | |
| **National/**  **state Level** | Ethiopia | India | Nepal | Nigeria | South Africa | Uganda |
|  |  |  |  | Yes | Yes |  |
| **District level** |  |  |  |  |  |  |
| **Source of Evidence/**  **Date for Data** |  |  |  |  |  |  |
| **Source of Q** |  |  |  |  |  |  |

**Table 10: 3.0 MENTAL HEALTH POLICY cont…**

| **Questions/**  **themes** | **3.2.0 Does the policy / strategy include:** | | | | | |
| --- | --- | --- | --- | --- | --- | --- |
| **3.2.4** | **Integration into general hospitals?** | | | | | |
|  | **Countries** | | | | | |
| **National/**  **state Level** | Ethiopia | India | Nepal | Nigeria | South Africa | Uganda |
|  | Yes | Not applicable |  |  |  |  |
| **District level** |  |  |  |  |  |  |
| **Source of Evidence/**  **Date for Data** |  |  |  |  |  |  |
| **Source of Q** |  |  |  |  |  |  |

**Table 11: 3.0 MENTAL HEALTH POLICY cont…**

| **Questions/**  **themes** | **3.2.0 Does the policy / strategy include:** | | | | | |
| --- | --- | --- | --- | --- | --- | --- |
| **3.2.5** | **Maternal mental health services based on the following indices:**   1. **availability of empowered health workers to assess and manage maternal mental health services** 2. **availability of drugs and sundries for perinatal care** 3. **Other required infrastructure (such as examination and admission rooms)** 4. **Key equipment/tools such as gloves, Delivery bed, Partograph, examination light Scissors, blade, cord, clamp, Needles and syringes)** | | | | | |
|  | **Countries** | | | | | |
| **National/**  **state Level** | Ethiopia | India | Nepal | Nigeria | South Africa | Uganda |
|  |  |  |  |  |  |  |
| **District level** |  |  |  |  |  |  |
| **Source of Evidence/**  **Date for Data** |  |  |  |  |  |  |
| **Source of Q** |  |  |  |  |  |  |

**Table 12: 3.0 MENTAL HEALTH POLICY cont…**

| **Questions/**  **themes** | **3.2.0 Does the policy / strategy include:** | | | | | |
| --- | --- | --- | --- | --- | --- | --- |
| **3.2.6** | **HIV mental health?** | | | | | |
|  | **Countries** | | | | | |
| **National/**  **state Level** | Ethiopia | India | Nepal | Nigeria | South Africa | Uganda |
|  |  |  |  |  |  |  |
| **District level** |  |  |  |  |  |  |
| **Source of Evidence/**  **Date for Data** |  |  |  |  |  |  |
| **Source of Q** |  |  |  |  |  |  |

**Table 13: 3.0 MENTAL HEALTH POLICY cont…**

| **Questions/**  **themes** | **3.2.0 Does the policy / strategy include:** | | | | | |
| --- | --- | --- | --- | --- | --- | --- |
| 3.2.7 | **Alcohol misuse?** | | | | | |
|  | **Countries** | | | | | |
| **National/**  **state Level** | Ethiopia | India | Nepal | Nigeria | South Africa | Uganda |
|  |  |  |  |  |  |  |
| **District level** |  |  |  |  |  |  |
| **Source of Evidence/**  **Date for Data** |  |  |  |  |  |  |
| **Source of Q** |  |  |  |  |  |  |

**Table 14: 3.0 MENTAL HEALTH POLICY cont…**

| **Questions/**  **themes** | **3.2.0 Does the policy / strategy include:** | | | | | |
| --- | --- | --- | --- | --- | --- | --- |
| **3.2.8** | **Epilepsy?** | | | | | |
|  | **Countries** | | | | | |
| **National/**  **state Level** | Ethiopia | India | Nepal | Nigeria | South Africa | Uganda |
|  |  |  |  |  |  |  |
| **District level** |  |  |  |  |  |  |
| **Source of Evidence/**  **Date for Data** |  |  |  |  |  |  |
| **Source of Q** |  |  |  |  |  |  |

**Table 15: 3.0 MENTAL HEALTH POLICY cont…**

| **Questions/**  **themes** | - - 1. **Do the policy / strategy explicitly address issues of equity? Describe in relation to the following:** | | | | | |
| --- | --- | --- | --- | --- | --- | --- |
| **3.3.1** | **Gender?** | | | | | |
|  | **Countries** | | | | | |
| **National/**  **state Level** | Ethiopia | India | Nepal | Nigeria | South Africa | Uganda |
|  |  |  |  |  |  |  |
| **District level** |  |  |  |  |  |  |
| **Source of Evidence/**  **Date for Data** |  |  |  |  |  |  |
| **Source of Q** |  |  |  |  |  |  |

**Table 16: 3.0 MENTAL HEALTH POLICY cont…**

| **Questions/**  **themes** | - - 1. **Do the policy / strategy explicitly address issues of equity? Describe in relation to the following:** | | | | | |
| --- | --- | --- | --- | --- | --- | --- |
| **3.3.2** | **Rural / urban residence** | | | | | |
|  | **Countries** | | | | | |
| **National/**  **state Level** | Ethiopia | India | Nepal | Nigeria | South Africa | Uganda |
|  |  |  |  |  |  |  |
| **District level** |  |  |  |  |  |  |
| **Source of Evidence/**  **Date for Data** |  |  |  |  |  |  |
| **Source of Q** |  |  |  |  |  |  |

**Table 17: 3.0 MENTAL HEALTH POLICY cont…**

| **Questions/**  **themes** | - - 1. **Do the policy / strategy explicitly address issues of equity? Describe in relation to the following:** | | | | | |
| --- | --- | --- | --- | --- | --- | --- |
| **3.3.3** | **Low socio-economic status** | | | | | |
|  | **Countries** | | | | | |
| **National/**  **state Level** | Ethiopia | India | Nepal | Nigeria | South Africa | Uganda |
|  |  |  |  |  |  |  |
| **District level** |  |  |  |  |  |  |
| **Source of Evidence/**  **Date for Data** |  |  |  |  |  |  |
| **Source of Q** |  |  |  |  |  |  |

**Table 18: 3.0 MENTAL HEALTH POLICY cont…**

| **Questions/**  **themes** | - - 1. **Do the policy / strategy explicitly address issues of equity? Describe in relation to the following:** | | | | | |
| --- | --- | --- | --- | --- | --- | --- |
| **3.3.4** | **Disability** | | | | | |
|  | **Countries** | | | | | |
| **National/**  **state Level** | Ethiopia | India | Nepal | Nigeria | South Africa | Uganda |
|  |  |  |  |  |  |  |
| **District level** |  |  |  |  |  |  |
| **Source of Evidence/**  **Date for Data** |  |  |  |  |  |  |
| **Source of Q** |  |  |  |  |  |  |

**Table 19: 3.0 MENTAL HEALTH POLICY cont…**

| **Questions/**  **themes** | - - 1. **Is there any specific provision for reaching vulnerable populations (especially the poor and those with severe mental disorder)? Describe** | | | | | |
| --- | --- | --- | --- | --- | --- | --- |
|  |  | | | | | |
|  | **Countries** | | | | | |
| **National/**  **state Level** | Ethiopia | India | Nepal | Nigeria | South Africa | Uganda |
|  |  |  |  |  |  |  |
| **District level** |  |  |  |  |  |  |
| **Source of Evidence/**  **Date for Data** |  |  |  |  |  |  |
| **Source of Q** |  |  |  |  |  |  |

**Table 20: 4.0 MENTAL HEALTH PLAN**

| **Questions/**  **themes** | - - 1. **Existence of an officially approved mental health policy and/or plan?** | | | | | |
| --- | --- | --- | --- | --- | --- | --- |
|  |  | | | | | |
|  | **Countries** | | | | | |
| **National/**  **state Level** | Ethiopia | India | Nepal | Nigeria | South Africa | Uganda |
|  |  |  |  |  |  |  |
| **District level** |  |  |  |  |  |  |
| **Source of Evidence/**  **Date for Data** |  |  |  |  |  |  |
| **Source of Q** |  |  |  |  |  |  |

**Table 21: 4.0 MENTAL HEALTH PLAN**

| **Questions/**  **themes** | - - 1. **Existence of an officially approved mental health policy and/or plan?** | | | | | |
| --- | --- | --- | --- | --- | --- | --- |
| **4.1.1.** | **If present, what year was mental health plan last revised?** | | | | | |
|  | **Countries** | | | | | |
| **National/**  **state Level** | Ethiopia | India | Nepal | Nigeria | South Africa | Uganda |
|  | Not applicable |  |  |  |  |  |
| **District level** |  |  |  |  |  |  |
| **Source of Evidence/**  **Date for Data** |  |  |  |  |  |  |
| **Source of Q** |  |  |  |  |  |  |

**Table 22: 4.0 MENTAL HEALTH PLAN**

| **Questions/**  **themes** | **4.1.0. Existence of an officially approved mental health policy and/or plan?** | | | | | |
| --- | --- | --- | --- | --- | --- | --- |
| **4.1.2.** | **If present, describe how much of the plan has been implemented? And across how much of the country / state / district?** | | | | | |
|  | **Countries** | | | | | |
| **National/**  **state Level** | Ethiopia | India | Nepal | Nigeria | South Africa | Uganda |
|  |  |  |  |  |  |  |
| **District level** |  |  |  |  |  |  |
| **Source of Evidence/**  **Date for Data** |  |  |  |  |  |  |
| **Source of Q** |  |  |  |  |  |  |

**Table 23: 4.0 MENTAL HEALTH PLAN**

| **Questions/**  **themes** | **4.2.0. Does the** Region/ **District Health plan include:** | | | | | |
| --- | --- | --- | --- | --- | --- | --- |
| **4.2.1.** | **Integration of mental health into PHC?** | | | | | |
|  | **Countries** | | | | | |
| **National/**  **state Level** | Ethiopia | India | Nepal | Nigeria | South Africa | Uganda |
|  |  |  |  |  |  |  |
| **District level** |  |  |  |  |  |  |
| **Source of Evidence/**  **Date for Data** |  |  |  |  |  |  |
| **Source of Q** |  |  |  |  |  |  |

**Table 24: 4.0 MENTAL HEALTH PLAN**

| **Questions/**  **themes** | **4.2.0. Does the** Region/ **District Health plan include:** | | | | | |
| --- | --- | --- | --- | --- | --- | --- |
| **4.2.2.** | **Maternal mental health?** | | | | | |
|  | **Countries** | | | | | |
| **National/**  **state Level** | Ethiopia | India | Nepal | Nigeria | South Africa | Uganda |
|  | No |  |  |  |  |  |
| **District level** |  |  |  |  |  |  |
| **Source of Evidence/**  **Date for Data** |  |  |  |  |  |  |
| **Source of Q** |  |  |  |  |  |  |

**Table 25: 4.0 MENTAL HEALTH PLAN**

| **Questions/**  **themes** | **4.2.0. Does the** Region/ **District Health plan include:** | | | | | |
| --- | --- | --- | --- | --- | --- | --- |
| **4.2.3.** | **HIV mental health?** | | | | | |
|  | **Countries** | | | | | |
| **National/**  **state Level** | Ethiopia | India | Nepal | Nigeria | South Africa | Uganda |
|  |  |  |  |  |  |  |
| **District level** |  |  |  |  |  |  |
| **Source of Evidence/**  **Date for Data** |  |  |  |  |  |  |
| **Source of Q** |  |  |  |  |  |  |

**Table 26: 4.0 MENTAL HEALTH PLAN**

| **Questions/**  **themes** | **4.2.0. Does the** Region/ **District Health plan include:** | | | | | |
| --- | --- | --- | --- | --- | --- | --- |
| **4.2.4.** | **Alcohol misuse?** | | | | | |
|  | **Countries** | | | | | |
| **National/**  **state Level** | Ethiopia | India | Nepal | Nigeria | South Africa | Uganda |
|  |  |  |  |  |  |  |
| **District level** |  |  |  |  |  |  |
| **Source of Evidence/**  **Date for Data** |  |  |  |  |  |  |
| **Source of Q** |  |  |  |  |  |  |

**Table 27: 4.0 MENTAL HEALTH PLAN**

| **Questions/**  **themes** | **4.2.0. Does the** Region/ **District Health plan include:** | | | | | |
| --- | --- | --- | --- | --- | --- | --- |
| **4.2.5.** | **Epilepsy?** | | | | | |
|  | **Countries** | | | | | |
| **National/**  **state Level** | Ethiopia | India | Nepal | Nigeria | South Africa | Uganda |
|  |  |  |  |  |  |  |
| **District level** |  |  |  |  |  |  |
| **Source of Evidence/**  **Date for Data** |  |  |  |  |  |  |
| **Source of Q** |  |  |  |  |  |  |

**Table 28: 4.0 MENTAL HEALTH PLAN**

| **Questions/**  **themes** | **4.3.0. Does the plan explicitly address issues of equity? Describe in relation to the following:** | | | | | |
| --- | --- | --- | --- | --- | --- | --- |
| **4.3.0.** | **Does the plan explicitly address issues of equity?** | | | | | |
|  | **Countries** | | | | | |
| **National/**  **state Level** | Ethiopia | India | Nepal | Nigeria | South Africa | Uganda |
|  |  |  |  |  |  |  |
| **District level** |  |  |  |  |  |  |
| **Source of Evidence/**  **Date for Data** |  |  |  |  |  |  |
| **Source of Q** |  |  |  |  |  |  |

**Table 29: 4.0 MENTAL HEALTH PLAN**

| **Questions/**  **themes** | **4.3.0. Does the plan explicitly address issues of equity? Describe in relation to the following:** | | | | | |
| --- | --- | --- | --- | --- | --- | --- |
| **4.3.1.** | **Gender** | | | | | |
|  | **Countries** | | | | | |
| **National/**  **state Level** | Ethiopia | India | Nepal | Nigeria | South Africa | Uganda |
|  |  |  |  |  |  |  |
| **District level** |  |  |  |  |  |  |
| **Source of Evidence/**  **Date for Data** |  |  |  |  |  |  |
| **Source of Q** |  |  |  |  |  |  |

**Table 30: 4.0 MENTAL HEALTH PLAN**

| **Questions/**  **themes** | **4.3.0. Does the plan explicitly address issues of equity? Describe in relation to the following:** | | | | | |
| --- | --- | --- | --- | --- | --- | --- |
| **4.3.2.** | **Rural / urban residence** | | | | | |
|  | **Countries** | | | | | |
| **National/**  **state Level** | Ethiopia | India | Nepal | Nigeria | South Africa | Uganda |
|  |  |  |  |  |  |  |
| **District level** |  |  |  |  |  |  |
| **Source of Evidence/**  **Date for Data** |  |  |  |  |  |  |
| **Source of Q** |  |  |  |  |  |  |

**Table 32: 4.0 MENTAL HEALTH PLAN**

| **Questions/**  **themes** | **4.3.0. Does the plan explicitly address issues of equity? Describe in relation to the following:** | | | | | |
| --- | --- | --- | --- | --- | --- | --- |
| **4.3.3.** | **Low socio-economic** status | | | | | |
|  | **Countries** | | | | | |
| **National/**  **state Level** | Ethiopia | India | Nepal | Nigeria | South Africa | Uganda |
|  |  |  |  |  |  |  |
| **District level** |  |  |  |  |  |  |
| **Source of Evidence/**  **Date for Data** |  |  |  |  |  |  |
| **Source of Q** |  |  |  |  |  |  |

**Table 33: 4.0 MENTAL HEALTH PLAN**

| **Questions/**  **themes** | **4.3.0. Does the plan explicitly address issues of equity? Describe in relation to the following:** | | | | | |
| --- | --- | --- | --- | --- | --- | --- |
| **4.3.4.** | **Disability** | | | | | |
|  | **Countries** | | | | | |
| **National/**  **state Level** | Ethiopia | India | Nepal | Nigeria | South Africa | Uganda |
|  |  |  |  |  |  |  |
| **District level** |  |  |  |  |  |  |
| **Source of Evidence/**  **Date for Data** |  |  |  |  |  |  |
| **Source of Q** |  |  |  |  |  |  |

**Table 34: 4.0 MENTAL HEALTH PLAN**

| **Questions/**  **themes** | **4.4. Does the plan have any specific provision for reaching vulnerable populations (especially the poor and those with severe mental disorder)? Describe** | | | | | |
| --- | --- | --- | --- | --- | --- | --- |
|  | **Countries** | | | | | |
| **National/**  **state Level** | Ethiopia | India | Nepal | Nigeria | South Africa | Uganda |
|  |  |  |  |  |  |  |
| **District level** |  |  |  |  |  |  |
| **Source of Evidence/**  **Date for Data** |  |  |  |  |  |  |
| **Source of Q** |  |  |  |  |  |  |

**Table 35: 4.0 MENTAL HEALTH PLAN**

| **Questions/**  **themes** | **4.5. Does the plan explicitly address issues of monitoring and evaluation?** | | | | | |
| --- | --- | --- | --- | --- | --- | --- |
|  | **Countries** | | | | | |
| **National/**  **state Level** | Ethiopia | India | Nepal | Nigeria | South Africa | Uganda |
|  |  |  |  |  |  |  |
| **District level** |  |  |  |  |  |  |
| **Source of Evidence/**  **Date for Data** |  |  |  |  |  |  |
| **Source of Q** |  |  |  |  |  |  |

**Table 36: 4.0 MENTAL HEALTH PLAN**

| **Questions/**  **themes** | **4.6. Does the national HMIS system capture mental health indicators?** | | | | | |
| --- | --- | --- | --- | --- | --- | --- |
|  | **Countries** | | | | | |
| **National/**  **state Level** | Ethiopia | India | Nepal | Nigeria | South Africa | Uganda |
|  |  |  |  |  |  |  |
| **District level** |  |  |  |  |  |  |
| **Source of Evidence/**  **Date for Data** |  |  |  |  |  |  |
| **Source of Q** |  |  |  |  |  |  |

**Table 37: 5.0. MENTAL HEALTH LEGISLATION**

| **Questions/**  **themes** | **5.1. Evidence of dedicated mental health legislation?** | | | | | |
| --- | --- | --- | --- | --- | --- | --- |
|  | **Countries** | | | | | |
| **National/**  **state Level** | Ethiopia | India | Nepal | Nigeria | South Africa | Uganda |
|  |  |  |  |  |  |  |
| **District level** |  |  |  |  |  |  |
| **Source of Evidence/**  **Date for Data** |  |  |  |  |  |  |
| **Source of Q** |  |  |  |  |  |  |

**Table 38: 5.0. MENTAL HEALTH LEGISLATION**

| **Questions/**  **themes** | **5.1. Evidence of dedicated mental health legislation?** | | | | | |
| --- | --- | --- | --- | --- | --- | --- |
| **5.1.1.** | **If present, year of last revision?** | | | | | |
|  | **Countries** | | | | | |
| **National/**  **state Level** | Ethiopia | India | Nepal | Nigeria | South Africa | Uganda |
|  |  |  |  |  |  |  |
| **District level** |  |  |  |  |  |  |
| **Source of Evidence/**  **Date for Data** |  |  |  |  |  |  |
| **Source of Q** |  |  |  |  |  |  |

**Table 39: 5.0. MENTAL HEALTH LEGISLATION**

| **Questions/**  **themes** | **5.1. Evidence of dedicated mental health legislation?** | | | | | |
| --- | --- | --- | --- | --- | --- | --- |
| **5.1.2.** | **If present, describe how much of the legislation is implemented, and in which geographical areas? (guidance, availability of personnel, monitoring)** | | | | | |
|  | **Countries** | | | | | |
| **National/**  **state Level** | Ethiopia | India | Nepal | Nigeria | South Africa | Uganda |
|  |  |  |  |  |  |  |
| **District level** |  |  |  |  |  |  |
| **Source of Evidence/**  **Date for Data** |  |  |  |  |  |  |
| **Source of Q** |  |  |  |  |  |  |

**Table 40: 5.0. MENTAL HEALTH LEGISLATION**

| **Questions/**  **themes** | **5.1. Evidence of dedicated mental health legislation?** | | | | | |
| --- | --- | --- | --- | --- | --- | --- |
| **5.1.3.** | **5. Any protocols for managing patients who require treatment against their will?** | | | | | |
|  | **Countries** | | | | | |
| **National/**  **state Level** | Ethiopia | India | Nepal | Nigeria | South Africa | Uganda |
|  |  |  |  |  |  |  |
| **District level** | None |  |  |  |  |  |
| **Source of Evidence/**  **Date for Data** |  |  |  |  |  |  |
| **Source of Q** |  |  |  |  |  |  |

**Table 41: 6.0. BENEFITS**

| **Questions/**  **themes** | **6.1. 0Are they available? Which illnesses? Who is eligible? Any benefits / welfare payments for persons with mental illness?** | | | | | |
| --- | --- | --- | --- | --- | --- | --- |
|  | **Countries** | | | | | |
| **National/**  **state Level** | Ethiopia | India | Nepal | Nigeria | South Africa | Uganda |
|  |  |  |  |  |  |  |
| **District level** |  |  |  |  |  | None |
| **Source of Evidence/**  **Date for Data** |  |  |  |  |  |  |
| **Source of Q** |  |  |  |  |  |  |

Table 42: **7.0. HUMAN RESOURCES**

| **Questions/themes** |  |  |  |  |
| --- | --- | --- | --- | --- |
| **7.1. Mental health professionals in the country, working in PUBLIC sector** | ***MH worker^[[1]](#footnote-2)^*** | **National / State level** | **District level** |  |
|  | ***Psychiatrists*** |  |  |  |
| Ethiopia |  | Addis Ababa: | Regional towns: |  |
| India |  |  |  |  |
| Nepal |  |  |  |  |
| Nigeria |  |  |  |  |
| South Africa |  |  |  |  |
| Uganda |  |  |  |  |
|  | **Neurologists** | **National / State level** | **District level** |  |
| Ethiopia |  |  |  |  |
| India |  |  |  |  |
| Nepal |  |  |  |  |
| Nigeria |  |  |  |  |
| South Africa |  |  |  |  |
| Uganda |  |  |  |  |
|  | **Psychiatric Nurses** | **National / State level** | **District level** |  |
| Ethiopia |  |  |  |  |
| India |  |  |  |  |
| Nepal |  |  |  |  |
| Nigeria |  |  |  |  |
| South Africa |  |  |  |  |
| Uganda |  |  |  |  |
|  | **Clinical psychologists** | **National / State level** | **District level** |  |
| Ethiopia |  |  |  |  |
| India |  |  |  |  |
| Nepal |  |  |  |  |
| Nigeria |  |  |  |  |
| South Africa |  |  |  |  |
| Uganda |  |  |  |  |
|  | **Social workers** | **National / State level** | **District level** |  |
| Ethiopia |  |  |  |  |
| India |  |  |  |  |
| Nepal |  |  |  |  |
| Nigeria |  |  |  |  |
| South Africa |  |  |  |  |
| Uganda |  |  |  |  |
|  | **Occupational therapist** |  |  |  |
| Ethiopia |  |  |  |  |
| India |  |  |  |  |
| Nepal |  |  |  |  |
| Nigeria |  |  |  |  |
| South Africa |  |  |  |  |
| Uganda |  |  |  |  |
|  | **others** | **National / State level** | **District level** |  |
| Ethiopia |  |  |  |  |
| India |  |  |  |  |
| Nepal | Trained nurses |  |  |  |
| Nigeria |  |  |  |  |
| South Africa |  |  |  |  |
| Uganda | Psychiatric Clinical Officers |  |  |  |
| **Note on these statistics**  “A comparison between human resource levels is complicated by differences in definitions of core mental health staff in the older and newer Atlas versions. For example, in 2005 the rate of **psychiatric nurses** was requested. In 2011, this measure was defined as the rate of nurses working in mental health. This change was made because in many LAMICs there are a number of nurses working in mental health facilities that do not have formal training to the same extent as psychiatric nurses. As such, in 2005 there may have been an underestimation of the availability of nurses for mental health. Likewise, the definition of social workers differed between the two assessments. Furthermore, while psychologists were defined in a similar manner in both Atlases, large and in many instances, implausible differences in rates for many countries between 2005 and 2011 suggests that countries may have difficulty providing accurate figures for this category of professionals”. :WHO ATLAS 2011 | | | | |

**Table 43: 7.0. HUMAN RESOURCES**

| **7.2. Mental health professionals in the country, working ONLY IN PRIVATE sector** | **MH worker^[[2]](#footnote-3)^** | **National / State level** | **District level** |  |
| --- | --- | --- | --- | --- |
|  | **Psychiatrists** |  |  |  |
| Ethiopia |  |  |  |  |
| India |  |  |  |  |
| Nepal |  |  |  |  |
| Nigeria |  |  |  |  |
| South Africa |  |  |  |  |
| Uganda |  |  |  |  |
|  | Neurologists | **National / State level** | **District level** |  |
| Ethiopia |  |  |  |  |
| India |  |  |  |  |
| Nepal |  |  |  |  |
| Nigeria |  |  |  |  |
| South Africa |  |  |  |  |
| Uganda |  |  |  |  |
|  | Psychiatric Nurses | **National / State level** | **District level** |  |
| Ethiopia |  |  |  |  |
| India |  |  |  |  |
| Nepal |  |  |  |  |
| Nigeria |  |  |  |  |
| South Africa |  |  |  |  |
| Uganda |  |  |  |  |
|  | Clinical Psychologists | **National / State level** | **District level** |  |
| Ethiopia |  |  |  |  |
| India |  |  |  |  |
| Nepal |  |  |  |  |
| Nigeria |  |  |  |  |
| South Africa |  |  |  |  |
| Uganda |  |  |  |  |
|  | Social Workers | **National / State level** | **District level** |  |
| Ethiopia |  |  |  |  |
| India |  |  |  |  |
| Nepal |  |  |  |  |
| Nigeria |  |  |  |  |
| South Africa |  |  |  |  |
| Uganda |  |  |  |  |
|  | Others (specify) | **National / State level** | **District level** |  |
| Ethiopia |  |  |  |  |
| India |  |  |  |  |
| Nepal |  |  |  |  |
| Nigeria |  |  |  |  |
| South Africa |  |  |  |  |
| Uganda | Psychiatric Clinical Officers |  |  |  |

1. Insert relevant categories for country [↑](#footnote-ref-2)
2. [↑](#footnote-ref-3)
